# Characterization of Ti/Cu Dissimilar Metal Butt-Welded by the Cold Welding Process

**DOI:** 10.3390/ma19010197

**Published:** 2026-01-05

**Authors:** Yunyi Xiao, Fei Liu, Nuo Chen

**Affiliations:** School of Materials Science and Engineering, Hefei University of Technology, Hefei 230009, China; xyy2347891@outlook.com (Y.X.); 15656380810@163.com (N.C.)

**Keywords:** cold welding, dissimilar metal (Cu/Ti) welding, microstructure, mechanical properties, Cu_3_Ti, diffusion, solidification thermodynamics

## Abstract

Titanium alloys and copper have broad applications in aerospace, defense, and industry, but their dissimilar welding faces challenges from significant physicochemical differences and easy formation of brittle Ti-Cu intermetallic compounds, while existing methods like laser welding or friction stir welding have limitations, such as low strength or inability to weld ultra-thin plates. This study adopted cold welding to join Ti-6.5Al-1Mo-1V-2Zr alloy and 99.90% pure copper. The mechanical properties of the joint were tested, the microstructure and fracture of the weld were observed, and the phase composition of the weld was analyzed. The results show that the weld fusion zone mainly consists of Cu-based solid solution and Cu_3_Ti. Low cold welding heat input reduces the Cu_3_Ti content, so the joint mechanical properties do not decrease significantly. The tensile strength of the joint reaches 284 MPa, which is 83% of that of copper-based metals, and the elongation rate reaches 6.25%. Diffusion kinetics and solidification thermodynamics analyses confirm that Cu_3_Ti intermetallic compounds are preferentially generated in the weld seam.

## 1. Introduction

Titanium and its alloys have the advantages of low density, high specific strength, strong corrosion resistance, and plastic deformation, which are widely used in the fields of defense, automobile manufacturing, and aerospace engineering [1,2,3]. Copper and its alloys have good features of electrical conductivity, thermal conductivity, corrosion resistance, and easy processing and forming, which are also widely used in the fields of defense, automobile manufacturing, and industrial production [4,5]. To realize the welding of titanium and copper dissimilar metals, the composite structure can not only make up for the deficiency of titanium thermal conductivity but also remedy the poor mechanical properties of copper and further improve the corrosion resistance of copper [6,7].

The physical and chemical properties of titanium and copper are significantly different. In particular, the differences in melting point, specific heat capacity, thermal conductivity, and linear expansion coefficient will cause high residual stress and large deformation of the weld seam and base material. In addition, titanium and copper elements have limited mutual solubility in the liquid state and low mutual solubility in the solid state, and Ti-Cu intermetallic compounds, such as Cu_3_Ti, Cu_4_Ti, and Cu_4_Ti_3_, are easily formed between them [8]. The combined effect of residual stress and intermetallic compounds makes the mechanical properties of the welded joint between titanium and copper very poor [9]. Therefore, there are some challenges in achieving high-quality Ti/Cu welding.

At present, Ti/Cu dissimilar material welding methods that have been reported by scientists include electron beam [10,11], laser welding [12,13], friction stir welding [14,15], and other welding methods [16,17,18,19]. The results show that a large number of Ti-Cu intermetallic compounds are generated in the weld seam when the electron or laser beam is placed at the interface of Ti/Cu. When the electron or laser beam is biased on the copper side, the number of intermetallic compounds generated in the interface is significantly reduced. The reduction in the number of Ti-Cu metal compounds can improve the mechanical properties of the Ti/Cu dissimilar material joint, while currently, when using welding methods with high energy density like electron beam welding and laser welding, the joint strengths achieved by adjusting process parameters are 147 MPa and 225 MPa, respectively, which still do not meet the application requirements. When friction welding is used to weld Ti/Cu dissimilar materials, the amount of Ti-Cu intermetallic compounds generated in the weld is reduced. Ti and Cu form a vortex-like biphasic metal mixed structure [3], and the tensile strength of the joint can reach 95% of the copper base metal, but the ultra-thin plate, with a thickness of less than 1 mm, is not easily welded by stirring friction welding.

In this study, cold welding is used to weld the dissimilar material of titanium and copper. Cold welding is a pulsed-arc micro-welding process using short-duration, high-current electrical pulses to deposit an electrode material on a metallic substrate. Due to the extremely low heat input of cold welding technology, it is expected to be applied in the welding of thin metal sheets [20,21]. In the welding of Ti/Cu dissimilar metal sheets, it is hoped that the formation of Ti-Cu intermetallic compounds in the weld can be reduced and the mechanical properties of joints can be improved by using this method. At present, there is no report of using cold welding to weld Ti/Cu dissimilar metals.

## 2. Materials and Methods

**Figure 1:**
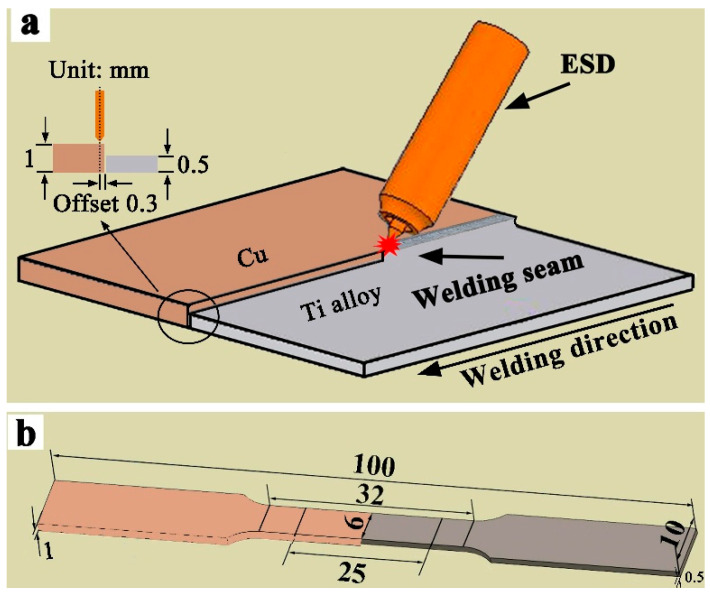
Schematic diagram of Ti/Cu dissimilar metal butt welding and tensile specimen size: (**a**) the schematic diagram of Ti/Cu dissimilar metal butt welding; (**b**) the tensile specimen size diagram.

Ti-6.5Al-1Mo-1V-2Zr alloy (The grade is TA15.) and copper (99.90%) were used for welding trials. The thicknesses of the Cu and Ti base metals were 1 mm and 0.5 mm, respectively, and the sizes were 50 mm × 100 mm. Before welding, we sanded the surface of the plate with sandpaper to remove the oxide film and wiped it with acetone to eliminate oil contaminants. In order to achieve a sufficient Cu melting amount during butt welding, the cold welding electrode was biased to the Cu side by 0.3 mm, and the welding schematic is shown in Figure 1a. After welding, the mechanical property test specimen was prepared according to the ASTM: E-8/E8M-2021 standard [22] and tensile experiments, and the shape and size of the specimen are shown in Figure 1b. The tensile speed was 3 mm/min, and the tensile strength of each parameter was taken as the average of 3 specimens. In addition, after the cross-section of the welded joint was cut by wire cutting, different types of sandpaper were prepared according to the standard metallographic sample preparation process. The specimen was then corroded with 15% phosphoric acid + 30% nitric acid + 55% glacial acetic acid for 25 s. The microscopic organization of the joint was observed by SEM, model JSM-6490LV, and the composition and phase detection in the welds used an electron probe micro-analyzer (EPMA), model EPMA-1600, and X-ray diffraction (XRD), model SmartLab SE.

## 3. Results and Discussion

### 3.1. Analysis of Surface Morphology and Microstructure of Welded Joints

**Figure 2:**
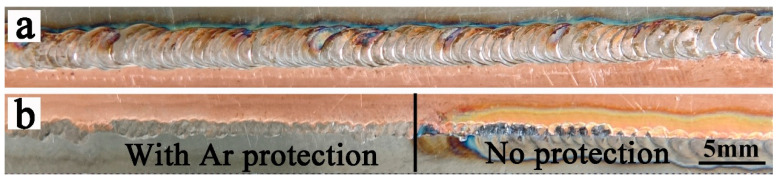
The surface morphology of the weld as observed by an optical microscope: (**a**) the front morphology of the weld; (**b**) the back morphology with and without argon protection.

Through repeated tests and optimization of welding parameters, it is found that the best welding parameters for 1 mm copper and 0.5 mm titanium alloy are a pulse current of 25 A, a pulse time of 20 ms, a welding speed of 400 mm/min, a front shielding gas flow rate of 5 L/min, and a back shielding gas flow rate of 3 L/min. Figure 2 is the surface morphology of the weld using the best welding parameters, Figure 2a is the upper surface of the weld, and Figure 2b is the back of the weld. It can be seen from Figure 2 that the surface of the weld is well formed, and there are relatively uniform fish scales, no splash, and no cracks. In general, when Ti alloy is welded, the surface needs to be covered, and argon gas is introduced to protect the surface of the weld from oxidation. When cold welding is used, the welding time is relatively short (20 milliseconds), and the heat input is low. Although the upper surface is not filled with argon gas to protect the weld, the titanium alloy side has almost no oxidation. However, the back side of the weld seam still needs protection. As shown in Figure 2b, the front half of the back of the weld is protected by argon, and neither titanium nor copper is oxidized; the second half is heavily oxidized due to a lack of protection. This is due to the fact that titanium and copper alloys also undergo a melting and solidification process despite the short welding time. Therefore, oxidation will still occur without any protection.

**Figure 3:**
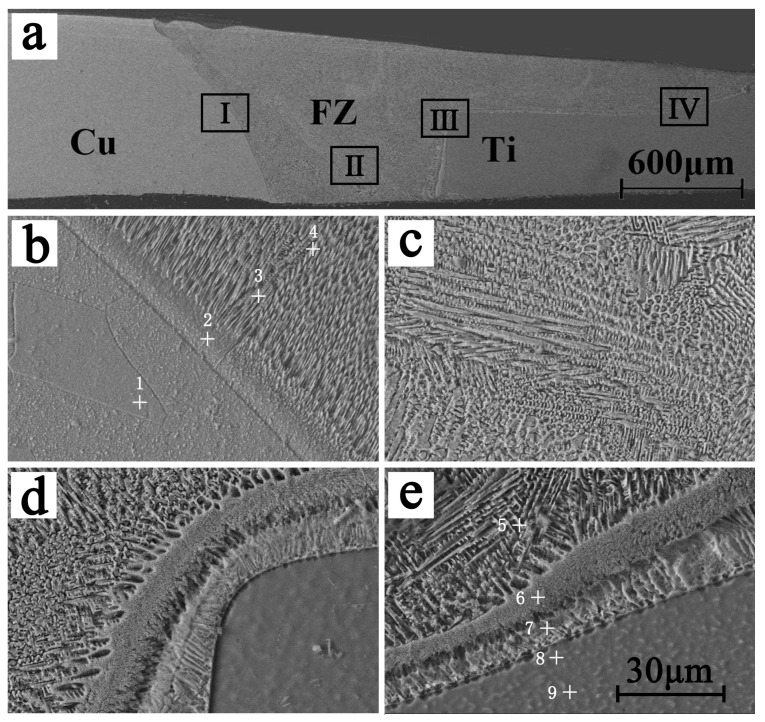
The microstructure of the weld seam. (**a**) is the macroscopic topography of the weld, and (**b**), (**c**), (**d**), and (**e**) are enlarged views of areas I, II, III, and IV in Figure 3a, respectively.

Figure 3 is the microstructure of the cross-section of the weld seam perpendicular to the weld direction. Figure 3a is the macroscopic topography of the weld, and Figure 3b–e are enlarged views of areas I, II, III, and IV in Figure 3a, respectively. It can be seen from Figure 3a that there are no obvious defects, such as porosity and cracks, in the weld, and the boundary between the base metal and the fusion area is clear. It can be inferred from the interface contour line between the titanium alloy base material and the fusion area that the titanium alloy base material is partially melted. It can be seen from Figure 3b–e that the fusion zone (FZ) is mainly composed of needle-like eutectic structures, which are very fine and uniform. The composition of the weld seam at different locations was tested using EPMA in order to study the composition and phase composition of different areas of the joint. The detection location is shown as the no. 1–9 marks in Figure 3b,e, and the composition results are shown in Table 1.

**Figure 4:**
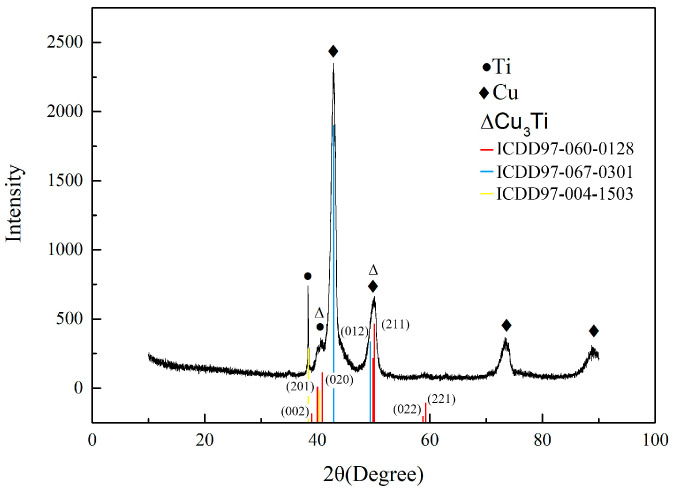
XRD diffraction pattern of the FZ.

It can be seen from Figure 3b that the grains of the Cu base metal near the FZ have grown significantly. There is a transition layer between the Cu base metal and the fusion zone. According to the analysis of its composition (position 2), the composition of the transition layer and the base metal composition of position 1 are basically the same, indicating that the transition layer has not undergone liquid–solid transformation. Figure 4 is the XRD diffraction pattern of the FZ. The needle-like structure in the FZ is the eutectic tissue of Cu and Cu_3_Ti, inferred from the composition of positions 3 and 4, combining the Cu-Ti phase diagram. It can be seen from Figure 3c that there is a partial branch crystal structure in the middle of the FZ, and the structure is relatively small. Figure 3d,e are the interfacial microstructure of the Ti base metal and the FZ. It can be seen that there are two transition layers between the Ti base metal and the FZ, and the thickness of the two transition layers is about 15 μm. The transition layers consist of Cu-based solid solutions and a small amount of Ti2Cu3 and TiCu4 based on the composition of positions 6 and 7.

**Figure 5:**
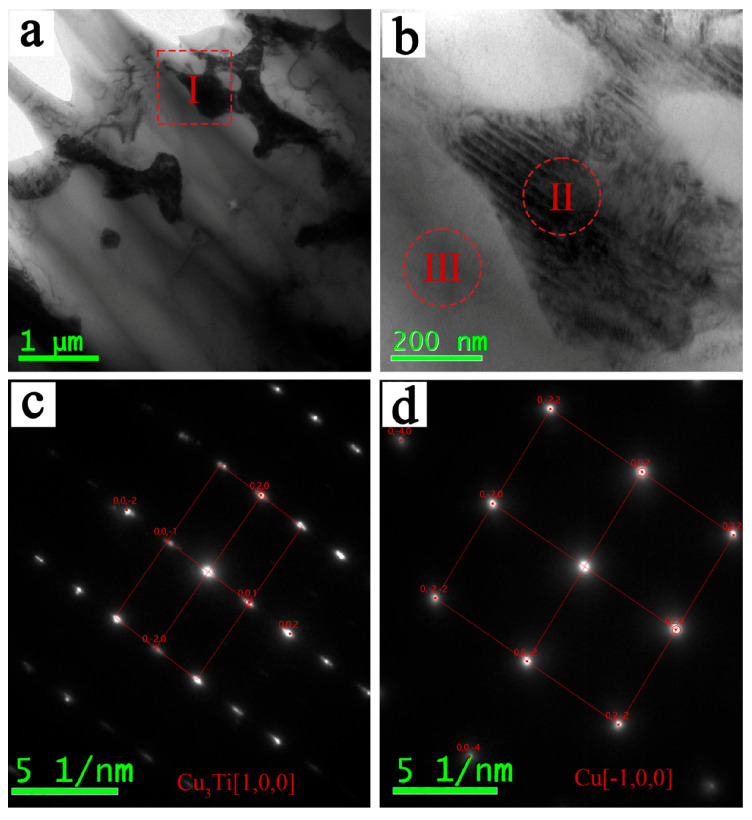
TEM images of the FZ: (**a**) bright field image of the FZ; (**b**) higher-magnification image of the red box in (**a**), (**c**), and (**d**) are the diffraction patterns of area II and area III in (**b**), respectively.

**Figure 6:**
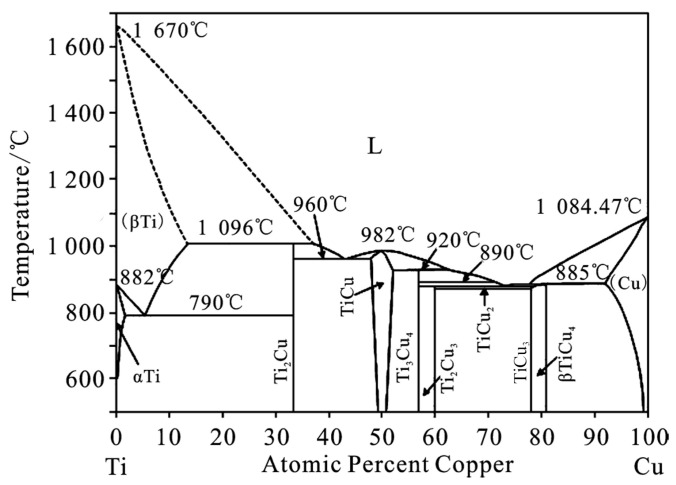
Binary equilibrium diagram of Ti/Cu.

It can be seen from Figure 3c that the microstructure in the FZ is relatively small. The welding joint was tested by a transmission electron microscope (TEM) to further study the phases contained in the FZ and their distribution positions; the results are shown in Figure 5. Figure 5a is the bright field image in the FZ, and Figure 5b is an enlarged view of the location of block I in Figure 5a. It can be seen from Figure 5a,b that the needle-like structure in the FZ is relatively small, with a size of 200−800 nm. Figure 5c,d are the diffraction patterns of area II and area III in Figure 5b, respectively. It can be seen from the analysis that the black area of position II is mainly composed of a Cu_3_Ti intermetallic compound, and the gray area of position III is composed of the Cu element. That is to say, the FZ is mainly composed of Cu and a small amount of Cu_3_Ti intermetallic compounds. The following is the detailed analysis:

Because the welding was carried out at a 0.3mm offset from the Cu side, and the thermal conductivity of Cu is higher, the heat diffusion on the Cu side is more complete, and the thickness of the Cu base material is greater than the thickness of the Ti base material; these make the Cu content in the FZ much higher than that of Ti. After XRD analysis and energy spectroscopy, the compound type at positions 3, 4, 5, and 6 in Figure 3 was mainly Cu_3_Ti. Although the weight ratio of Cu to Ti in the FZ exceeds 9:1, the good thermal conductivity of Cu and the small number of Ti atoms cause a large amount of Cu to precipitate out as solid solution before it has time to react with Ti to form a compound, resulting in the formation of Cu_3_Ti instead of Cu_4_Ti. In Figure 6, it can be seen that Cu_3_Ti precipitates at around 1213 K, so the Cu_3_Ti in the weld is first precipitated as a solid solution. The Cu_3_Ti in the weld first adheres to the Cu solid solution surface as a liquid phase, then precipitates gradually as the weld temperature decreases. At position 7, the proximity to the Ti parent material makes the number of Ti atoms more numerous and more uniformly distributed, thus allowing more Cu to react with Ti, so the main compound organization at point 7 is TiCu_4_.

### 3.2. Mechanical Properties and Fracture Analysis of Joints

**Figure 7:**
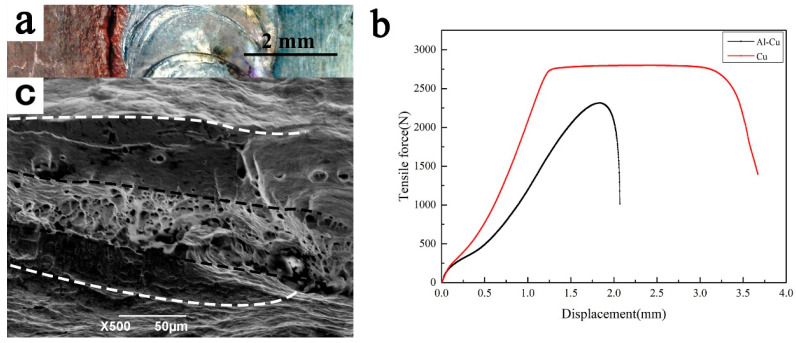
The property test of butt-welded Ti/Cu dissimilar metal: (**a**) the fracture location of the joint; (**b**) the tensile strength of Cu base material and joints; (**c**) the fracture surface of butt-welded Ti/Cu dissimilar metal.

Figure 7 is the mechanical properties test diagram and joint fracture position of the butt-welded Ti/Cu dissimilar metal. It can be seen from Figure 7a that the fracture occurs on the Cu base metal close to the weld seam. The reason may be that the grains of the base metal have grown due to the proximity to the weld seam, as shown in Figure 3b. The following is the detailed analysis:

The high thermal conductivity of Cu causes the base material in the heat-affected zone on the Cu side to grow by heat, resulting in a reduction in its plasticity. The Ti side of the heat-affected zone is also subject to heat growth, but because of the poor thermal conductivity of Ti, the location near the center of the weld melts and becomes the weld zone. The distance has slightly less heat absorption, and thus, the degree of grain growth is relatively small, in addition to the high tensile strength of the Ti base material itself, thus making the Cu side of the heat-affected zone become the weakest position in the mechanical properties of the specimen. This makes the Cu side of the heat-affected zone the weakest location in the specimen in terms of mechanical properties.

However, the grain growth is not obvious due to the low welding line energy, and the maximum tensile force of the joint reaches 2314 N, as shown in Figure 7b by the black curve. After multiple tests, the average tensile strength of the joint is 284 MPa, reaching 83% of the Cu base metal, even though the lowest tensile strength is still 278 MPa, reaching 81% of the Cu base metal. The elongation of the joint is 6.25%, while the elongation of the copper base material is 15%. Compared with the Cu base material, the elongation of the joint decreased by 58.3%, which may be due to the grain growth in the heat-affected zone of the Cu base material. The tensile strength of the joint was significantly improved compared with 151 MPa welded by laser melt brazing [23].

Figure 7c is the fracture surface of butt-welded Ti/Cu dissimilar metal. The fracture surface, outside the two white dotted lines in Figure 7c, is the slip band produced by dislocation slippage, which is due to the fact that the grain boundary slides to the surface when the specimen is subjected to tensile stress. As the tensile test progresses, cracks form on the surface of the specimen when the specimen is deformed to a certain extent, which, in turn, rapidly expands, forming an extended zone located between the white and black dotted lines. The area between the two black curves is the fracture zone; from the large number of ligaments present, it can be inferred that plastic deformation occurred before the joint broke.

### 3.3. Formation Mechanism of Cu_3_Ti in the Weld

To elucidate the fundamental reasons why Cu_3_Ti becomes the dominant intermetallic compound (IMC) in the cold-welded joint, diffusion kinetics calculations and solidification thermodynamics models are combined [24], and an estimation analysis is performed based on the actual conditions of cold welding. As can be seen from the data in Table 1, within the region of approximately 2300 μm covering points 3, 4, 5, and 6 in the FZ of Figure 3, the Ti content ranges from 5.11 wt.% to 5.58 wt.%, whereas in the narrow span of about 60 μm across points 8, 7, 6, and 5, the Ti content drops sharply from 89.1 wt.% to 5.93 wt.%. This is primarily due to the short duration of the molten pool during cold welding and the brief pulse time (20 ms), which leaves insufficient time for atoms to diffuse adequately. According to Fick’s second law:

(1) $$x=\sqrt{2Dt}$$

where x is the diffusion distance, D the diffusion coefficient, and t the diffusion time.

Based on the investigation of diffusion in the Cu–Ti system by Iijima et al. [25], the diffusion coefficient of Ti in solid Cu can be expressed by the Arrhenius equation:

(2) $$D_{\frac{Ti}{Cu}}=(0.693_{-0.135}^{+0.169})\times 10^{-4}exp[-\left(196\pm 2\right)\;kJ\;{mol}^{-1}/RT]m^{2}/s$$

where R is the gas constant (≈8.314 J·K^−1^·mol^−1^) and T is the absolute temperature (K).

Here, it is assumed that the diffusion coefficient in the liquid state is generally about five orders of magnitude higher than that in the solid state. Under the welding conditions, the diffusion coefficient is approximated as:

(3) $$\begin{array}{c} D\approx 1.4\times 10^{-7}\;m^{2}\;s^{-1} \end{array}$$

Given the dwell time above the transformation temperature of about 20 ms, taking the effective diffusion time t ≈ 20 ms and D into Equation (1) yields:

(4) $$x=\sqrt{2\times 1.4\times 10^{-7}\times 0.02}\approx 74.83\;\mu m$$

This result shows excellent agreement with the experimentally observed rapid decrease in Ti content over a distance of about 60 μm, confirming that Ti atom diffusion is highly restricted under cold welding conditions.

Because of the low heat input in cold welding, solidification proceeds rapidly. During the solidification of the FZ, Cu precipitates as the primary phase, and Cu_3_Ti begins to form in the final stages of solidification.

To approximately estimate the Ti concentration in the interdendritic liquid at the end of solidification, the Scheil–Gulliver model [26] is applied, which assumes complete diffusion in the liquid and negligible diffusion in the solid. The following equations relate the solid fraction $f_{S}$, solute content in the liquid $C_{L}$, and solute content in the solid $C_{S}$:

(5) $$C_{L}\left[wt\%\right]=C_{0}{\left(1-f_{S}\right)}^{k-1}$$

(6) $$C_{S}\left[wt\%\right]=k\cdot C_{0}{\left(1-f_{S}\right)}^{k-1}$$

where $C_{0}$ is the initial alloy concentration and k is the partition coefficient at a given temperature (defined as $C_{L}/C_{s}$).

From the phase diagram, k = 0.32. According to Table 1, the atomic ratio is Cu 91.48 at.% and Ti 8.52 at.%. The solid fraction $f_{S}$ is estimated as follows.

Considering the mass balance of Ti in the weld structure:

(7) $$m_{Ti}=m_{Ti\;dissolved\;in\;Cu}+m_{Ti\;in\;IMC}$$

The mass-conservation equation can be written as:

(8) $$f_{Cu}C_{Cu}+\left(1-f_{Cu}\right)C_{IMC}=C_{0}$$

where $f_{Cu}$ is the mass fraction of primary Cu, $\left(1-f_{Cu}\right)$ is the total mass fraction of the IMC phase, $C_{Cu}$ is the average Ti content (wt.%) in the primary Cu, $C_{IMC}$ is the average Ti content (wt.%) in the IMC, and $C_{0}$ = 6.45% is the initial Ti content of the alloy.

Thus,

(9) $$f_{Cu}=\frac{C_{IMC}-C_{0}}{C_{IMC}-C_{Cu}}$$

Since the IMC forms from Ti-enriched liquid at the end of solidification, $f_{Cu}$ is approximately equal to the solid fraction $f_{S}$.

The estimation is based on three considerations: (1) from the phase diagram, the equilibrium partition coefficient of Ti in Cu, $k_{0}$, is about 0.32, and the core composition of dendrites is approximately 2.7 wt.% Ti; (2) the equilibrium solid-solubility limit of Ti in Cu is about 4–5 wt.%; and (3) under rapid cooling, solid-state diffusion is completely suppressed, leading to a significant composition gradient from the core to the periphery of dendrites, with an overall average necessarily higher than the core value but limited by the solid-solubility limit. Accordingly, we take $C_{Cu}=3.0∼4.0\;wt.\%$ as a reasonable range.

The resulting atomic ratio range is $23.32\;at.\%<C_{L}<27.73\;at.\%$. It can be seen that the estimated atomic ratio is close to the 25 at.% Ti in Cu_3_Ti and 20 at.% in Cu_4_Ti, whereas it differs significantly from the required atomic ratios of other IMCs, such as Cu_2_Ti (33.3 at.% Ti), Cu_3_Ti_4_ (57.14 at.% Ti), and Cu_2_Ti_3_ (48.5 at.% Ti).

However, the reason why only Cu_3_Ti forms in the main region of the FZ during weld solidification, while Cu_4_Ti is not detected, can be proposed as follows:

From the perspective of nucleation thermodynamics, Cu_3_Ti belongs to the orthorhombic crystal system with space group Pmmn. Each Ti atom is surrounded by 12 Cu atoms, forming a TiCu_12_ cuboctahedron with only two inequivalent Cu sites. Its atomic arrangement exhibits relatively high symmetry and lower ordering difficulty. The Ti–Cu bond distances (2.55–2.69 Å) align well with the atomic spacing in the Cu matrix (~2.56 Å) [27], resulting in a low interfacial mismatch and small interfacial energy when nucleating on the Cu-based solid solution.

In contrast, although Cu_4_Ti is also orthorhombic (Pnma space group), it possesses four inequivalent Cu sites. The TiCu_12_ cuboctahedron must share vertices with 18 CuTi_2_Cu_10_ cuboctahedra, leading to significantly higher spatial complexity in the atomic arrangement [28]. Nucleation of Cu_4_Ti requires precise bonding of Ti atoms with Cu atoms in four distinct coordination environments, involving coordinated rearrangement of more atoms. Under the non-equilibrium conditions of rapid solidification, the thermal energy of atoms is insufficient to overcome the high nucleation barrier of Cu_4_Ti, completely suppressing its nucleation.

Finally, during solidification, Cu_3_Ti preferentially nucleates at the interdendritic regions of Cu at 1213 K, followed by rapid growth. It quickly occupies the remaining interdendritic liquid, thereby suppressing the precipitation of Cu_4_Ti. Consequently, Cu_3_Ti becomes the dominant intermetallic compound in the weld, while the formation of Cu_4_Ti is completely inhibited.

### 3.4. Discussion

This study demonstrates that cold welding effectively joins Ti-6.5Al-1Mo-1V-2Zr alloy to pure copper, overcoming the common challenge of excessive brittle intermetallic compound (IMC) formation reported in fusion-based methods, such as laser and electron beam welding. The joint tensile strength reached 284 MPa, significantly higher than the 147 MPa reported for laser melt brazing and comparable to friction stir welding, while also enabling the welding of thin plates (<1 mm), a notable limitation of the latter. These results confirm the initial hypothesis that low heat input and rapid solidification in cold welding can suppress the formation of multiple harmful IMCs.

Microstructural analysis revealed that the fusion zone consists primarily of Cu and Cu_3_Ti in a fine eutectic structure, with other IMCs largely absent. This selective formation of Cu_3_Ti is explained through integrated diffusion kinetics and solidification thermodynamics. The short pulse time (20 ms) limits Ti diffusion, creating a steep composition gradient. Scheil–Gulliver modeling indicates that the Ti concentration in the interdendritic liquid (23.32–27.73 at.%) closely matches the stoichiometry of Cu_3_Ti (25 at.% Ti), thermodynamically favoring its formation, while kinetic factors, such as its simpler crystal structure, further promote its nucleation under rapid cooling.

The fracture located in the Cu-side heat-affected zone (HAZ) rather than the fusion zone confirms that the tailored microstructure is not the weak point, shifting the performance constraint to the base metal’s thermal response—a more common and addressable issue in welding.

However, this work has limitations. The analysis is based on a single parameter set; therefore, a broader parametric study is needed. The models used are simplified, and long-term performance under fatigue or corrosive conditions remains unexamined. Future research should explore the process parameter window, employ advanced microstructure modeling, investigate post-weld treatments, and examine whether this kinetic control strategy can be applied to other dissimilar metal systems prone to brittle IMCs.

## 4. Conclusions

The dissimilar materials Ti and Cu were butt-welded successfully by cold welding. The major conclusions of this study can be summarized as follows:

(1) The weld surface is well formed, and there are no obvious defects. Due to the relatively low welding line energy, the front of the weld does not require a drag cover and argon protection, and the titanium alloy surface is not significantly oxidized. However, because the weld has undergone a liquid–solid transformation, the back side still needs protection.

(2) There are two transition layers with a thickness of about 15 μm between the Ti base metal and the FZ. The FZ mainly contains Cu elements, and the Ti base metal has a small amount of melting. The transition layer and fusion region are mainly composed of Cu-based solid solutions and eutectic tissues of Cu and Ti-Cu compounds, and the microstructure is needle-like and relatively small.

(3) The maximum tensile strength of the joint is 284 MPa, reaching 83% of the Cu base metal, and the fracture occurs on the Cu base metal near the weld because the grain in this part is slightly grown. The performance of the joint is significantly improved compared with other hot melt welding, such as laser welding.

(4) The preferential formation of Cu_3_Ti in the weld is attributed to suppressed atomic diffusion and rapid solidification under cold welding conditions, which thermodynamically and kinetically favor the nucleation of Cu_3_Ti over other Ti–Cu intermetallic compounds.

## Figures and Tables

**Table 1:** Chemical compositions of different areas detected using EPMA.

Serial Number	Percentage Composition (wt. %)					Inference composition
	Cu	Ti	Al	Zr	V	
1	99.66	-	0.34	-	-	Cu
2	99.06	-	0.94	-	-	Cu
3	93.24	5.11	1.31	0.09	0.25	(Cu), Cu_3_Ti
4	92.05	6.45	1.15	0.13	0.22	(Cu), Cu_3_Ti
5	91.50	5.93	2.03	0.08	0.46	(Cu), Cu_3_Ti
6	89.64	5.58	3.95	0.24	0.59	(Cu), Cu_3_Ti
7	80.83	15.12	2.66	0.15	1.24	(Cu), TiCu_4_
8	0.12	89.10	5.51	0.06	5.21	(Ti), TiAl_3_
9	0.08	91.54	6.38	-	2.01	(Ti), TiAl_3_

Note: (Cu) and (Ti) in the table represent Cu-based and Ti-based solutions, respectively.

## Data Availability

The original contributions presented in this study are included in the article. Further inquiries can be directed to the corresponding author.

## Abbreviations

The following abbreviations are used in this manuscript:

ASTM	American Society for Testing and Materials
SEM	Scanning Electron Microscopy
EPMA	Electron Probe Micro-Analyzer
XRD	X-ray Diffraction
TEM	Transmission Electron Microscopy
FZ	Fusion Zone
IMC	Intermetallic Compound
HAZ	Heat-Affected Zone
